# Tumor necrosis factor-alpha gene polymorphisms and susceptibility to ischemic heart disease: A systematic review and meta-analysis

**DOI:** 10.1097/MD.0000000000006569

**Published:** 2017-04-07

**Authors:** Peng Zhang, Xiaomei Wu, Guangxiao Li, Qiao He, Huixu Dai, Cong Ai, Jingpu Shi

**Affiliations:** Department of Clinical Epidemiology and Center of Evidence-Based Medicine, Institute of Cardiovascular Disease, The First Hospital of China Medical University, Shenyang, China.

**Keywords:** ischemic heart disease, meta-analysis, polymorphism, tumor necrosis factor-alpha

## Abstract

**Background:**

A number of studies had reported the association between tumor necrosis factor-alpha (TNF-α) gene polymorphisms and ischemic heart disease (IHD) risk. However, the results remained controversial. Therefore, we performed a systematic review with multiple meta-analyses to provide the more precise estimations of the relationship.

**Methods:**

We systematically searched electronic databases (PubMed, the Web of Science, EMBASE, Medline, Chinese National Knowledge Infrastructure, WanFang and ChongQing VIP Database) for relevant studies published up to February 2017. The odds ratios (ORs) and 95% confidence intervals (CIs) were estimated for assessing the association. The present meta-analysis was performed using STATA 12.0 software.

**Results:**

In total, 45 articles with 17,375 cases and 15,375 controls involved were included. Pooled ORs revealed a significant association between TNF-α −308G/A gene polymorphism and IHD (A vs. G: OR = 1.22, 95% CI = 1.10–1.35; (AA + GA) vs. GG: OR = 1.18, 95% CI = 1.03–1.36; (AA vs. (GA+GG): OR = 1.37, 95% CI = 1.08–1.75)), indicating that the TNF-α −308A allele might be an important risk factor for IHD. No association between other TNF-α gene polymorphisms and susceptibility to IHD were observed. No publication bias were found. Sensitivity analyses indicated that our results were stable.

**Conclusion:**

The present study indicated a possible association between the TNF-α −308G/A gene polymorphism and IHD risk. However, evidence was limited to confirm the role of TNF-α −238G/A, −857C/T, −863C/A, −1031T/C and other TNF-α gene polymorphisms in the risk of IHD.

## 1 Introduction

Ischemic heart disease (IHD) was known as one of the major burden to the healthcare system for not only cost but also death and disability worldwide.^[1]^ According to the estimation of the Global Burden of Disease Study in 2010, IHD was at the first place of the disability-adjusted life years (DALYs) ranking list for 291 diseases and injuries, accounting for 1884 DALYs per 100,000 population.^[1]^ Generally, myocardial infarction (MI), unstable/stable angina (UA/SA), and coronary artery disease (CAD) were regarded as the main cardiovascular types of IHD.^[2]^ The major defining pathologic feature of IHD was atherosclerosis, and proinflammatory and inflammatory factors contributing to the process of atherosclerosis were thought to play an important role in the pathogenesis of IHD.^[3]^ Proinflammatory cytokine tumor necrosis factor-alpha (TNF-α), which was produced by inflammatory cells like monocytes, macrophages, and neutrophils, could stimulate cytokine secretion and augment the inflammatory response in turn.^[4]^ All these processes were involved in the formation, progression, and rupture of the atherosclerotic plaque, thus, changed expression of TNF-α might make great contributions to the process of IHD.^[5]^

The TNF-α gene was located on chromosome 6p21.3 in human and arranged within the class III region of the major histocompatibility complex (MHC).^[6]^ Different polymorphisms of the TNF-α gene might cause different changes in the plasma level of TNF-α and take different effects in the course of IHD. To date, since the defect of the TNF-α gene was first studied in 1998,^[7]^ a large number of studies about the association between the TNF-α gene polymorphisms and IHD risk had been reported. Most studies were on the gene polymorphisms of TNF-α −238G/A (rs361525), −308G/A (rs1800629), −857C/T (rs1799724), −863C/A (rs1800630), and −1031T/C (rs1799964), while several studies were on the position of −376, −806, +476, +691, respectively.^[8,9]^ Some studies demonstrated that the TNF-α gene polymorphisms could change the susceptibility to IHD.^[10,11]^ However, other studies failed to confirm this relationship.^[12,13]^

Therefore, in the present study, we performed a systematic review with multiple meta-analyses aiming to draw a reliable conclusion on the overall association between the TNF-α gene polymorphisms and susceptibility to IHD.

## 2 Methods

We used computer-based literature search strategy to identify potential studies that evaluated the association between TNF-α gene polymorphisms and IHD risk. The present study were reported in accordance with the Preferred Reporting Items for Systematic Reviews and Meta-analysis (PRISMA) statement.^[14]^

### 2.1 Search strategy

Electronic literature databases including PubMed, the Web of Science, EMBASE, Medline, Chinese National Knowledge Infrastructure, WanFang and ChongQing VIP Database were searched independently by 2 reviewers for relevant studies published up to February 2017 without restrictions on language or type of document. The following search terms were used: (“TNF” OR “tumor necrosis factor”) AND (“polymorphism” OR “genotype” OR “variant” OR “mutation”) AND (“coronary artery disease” OR “angina” OR “myocardial infarction” OR “coronary heart disease” OR “ischemic heart disease” OR “ischemic cardiovascular disease”). In addition, references of the selected publications or textbooks were searched manually as a source of relevant studies. The approval by an ethics committee is not required because the present study was based on published studies.

### 2.2 Inclusion criteria

According to the inclusion criteria, 2 reviewers screened the relevant articles independently. Studies that met the following criteria were included in this meta-analysis: adult patients which were diagnosed with IHD; estimated the association between TNF-α gene polymorphisms and IHD risk; only cohort or case–control studies were included; had usable data on each genotype of both cases and controls. For reports on the same population or overlapping data, only the one with the largest sample size was included.

### 2.3 Data extraction and quality assessment

Information extraction of each eligible study was performed independently by 2 reviewers and disagreements were settled by a third reviewer. The following information were extracted from all eligible studies: the first author's name, publication year, study location, country, type of patient, ethnicity, source of control, genotyping method, study design, matching method between case group and control group, sample size together with percent of female, genotype distribution in both groups and Hardy–Weinberg equilibrium (HWE) evidence.

In accordance with the Newcastle-Ottawa Quality Assessment Scale (NOS),^[15]^ the quality assessment of all included studies were performed by 2 reviewers independently. Any disagreement was resolved by a third reviewer. The scores of each study ranged between 1 and 9, and studies with the scores >6 were recognized as of high quality.

### 2.4 Statistical analysis

The genotype frequencies in controls of each study were tested by Chi-square test for HWE.

The odds ratios (ORs) together with 95% confidence intervals (CIs) were used to estimate the association between TNF-α gene polymorphisms and susceptibility to IHD. Pooled ORs and 95% CIs were evaluated mainly according to 3 models which included allele model (B vs A), dominant model ((BB + AB) vs AA) and recessive model (BB vs (AB + AA)), respectively (the major allele was regarded as A and the minor allele as B).

*I*^2^ test was performed for evidence of heterogeneity among the results of different studies.^[16]^*I*^2^ > 50% was considered as a sign of significant heterogeneity using the random-effects model, and *I*^2^ ≤ 50% was considered nonheterogeneity using the fixed-effects model.^[17]^ Subgroup analyses were conducted to find the potential sources for heterogeneity, based on ethnicity, quality, HWE, control source, matching method, sample size, and genotyping method.

Begg test^[18]^ and Egger test^[19]^ was performed for the assessment of the potential publication bias. Sensitivity analysis was conducted by removing one study each time. *P* < .05 were regarded as statistically significant in all analyses. All statistical analyses were performed using the software STATA 12.0 (STATA, College Station, TX).

## 3 Results

### 3.1 Study selection

**Figure 1:**
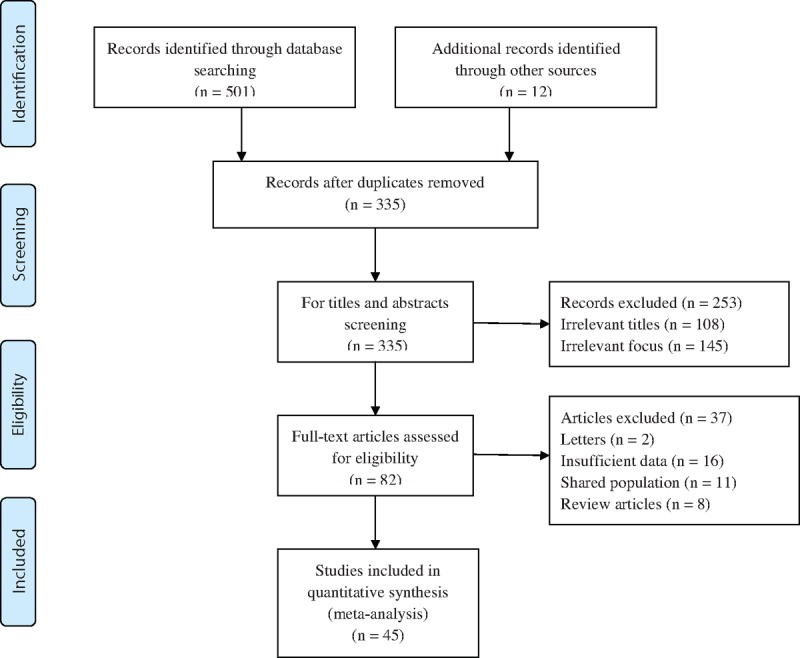
Flowchart of study selection.

A detailed description of the process of study selection is shown in Fig. 1. At first, 513 potential articles were identified from the databases searching, and only 335 articles remained after duplicating articles removed. After title and abstract screening, 253 irrelevant articles were excluded, and a total of 82 articles were fully reviewed. Finally, 45 articles (35 English studies and 10 Chinese studies) providing data for 17,375 cases and 15,375 controls were included in the present meta-analysis.

### 3.2 Study characteristics

The main characteristics of the included studies are shown in Table 1. Genotype deviation of HWE in controls was tested, and the results showed that most studies included were well designed. Two main sources of controls were observed, including hospital-based and population-based controls. The main genotyping method was polymerase chain reaction-restriction fragment length polymorphism (PCR-RFLP), and the main matching methods between cases and controls were age, sex, area, and ethnicity. Thirteen articles (including 15 independent studies) providing data for 5599 cases and 5863 controls were about the association between TNF-α −238G/A gene polymorphism and IHD risk, while 37 articles (including 44 independent studies) providing data for 15,849 cases and 13,782 controls were on TNF-α −308G/A gene polymorphism. The number of studies for gene polymorphisms of TNF-α −857C/T, −863C/A, and −1031T/C were 8, 13, and 7, respectively. In addition, there was one study on each position of −376, −806, +476, +691, respectively. However, due to the limited number of study, these 4 polymorphisms were not pooled.

**Table 1:**
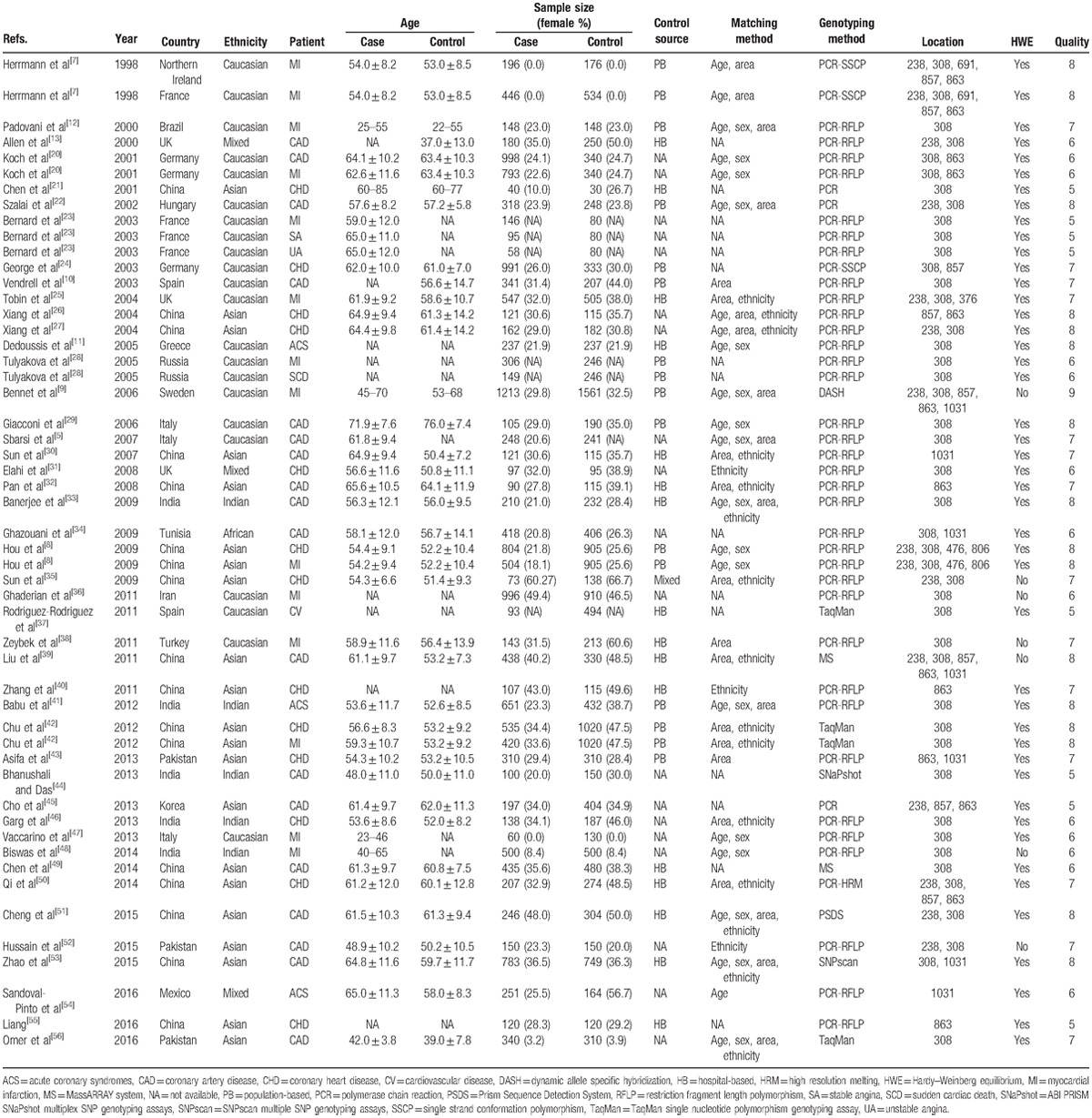
Main characteristics of included studies.

### 3.3 Association between TNF-α gene polymorphisms and IHD

#### 3.3.1 TNF-α −238G/A

**Figure 2:**
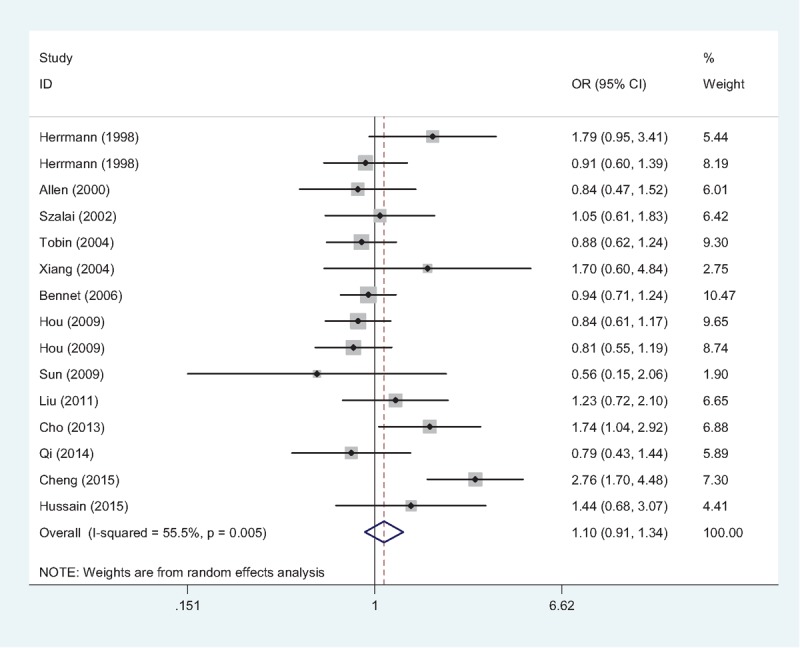
Forest plot of association between TNF-α −238G/A gene polymorphism (A vs G) and IHD risk. The association was indicated as OR with the corresponding 95% CI. The analysis was performed using the STATA 12.0. CI = confidence interval, IHD = ischemic heart disease, OR = odds ratio, TNF-α = tumor necrosis factor-alpha.

According to the results of heterogeneity test (A vs G: *P* = .005, *I*^2^ = 55.5%; (AA + GA) vs. GG: *P* = 0.002, *I*^2^ = 59.9%; AA vs (GA + GG): *P* = .555, *I*^2^ = 0.0%), random-effects models were used in allele model and dominant model, while fixed-effects model was used in the recessive model. The overall ORs demonstrated that there was no statistical association between TNF-α −238G/A gene polymorphism and IHD risk in neither genetic model (A vs G: OR = 1.10, 95% CI = 0.91–1.34, Fig. 2; (AA + GA) vs. GG: OR = 1.11, 95% CI = 0.90–1.38; AA vs (GA + GG): OR = 1.23, 95% CI = 0.69–2.21). Subgroup analyses were conducted; however, none of the pooled ORs achieved any statistical significance (data not shown).

#### 3.3.2 TNF-α −308G/A

**Figure 3:**
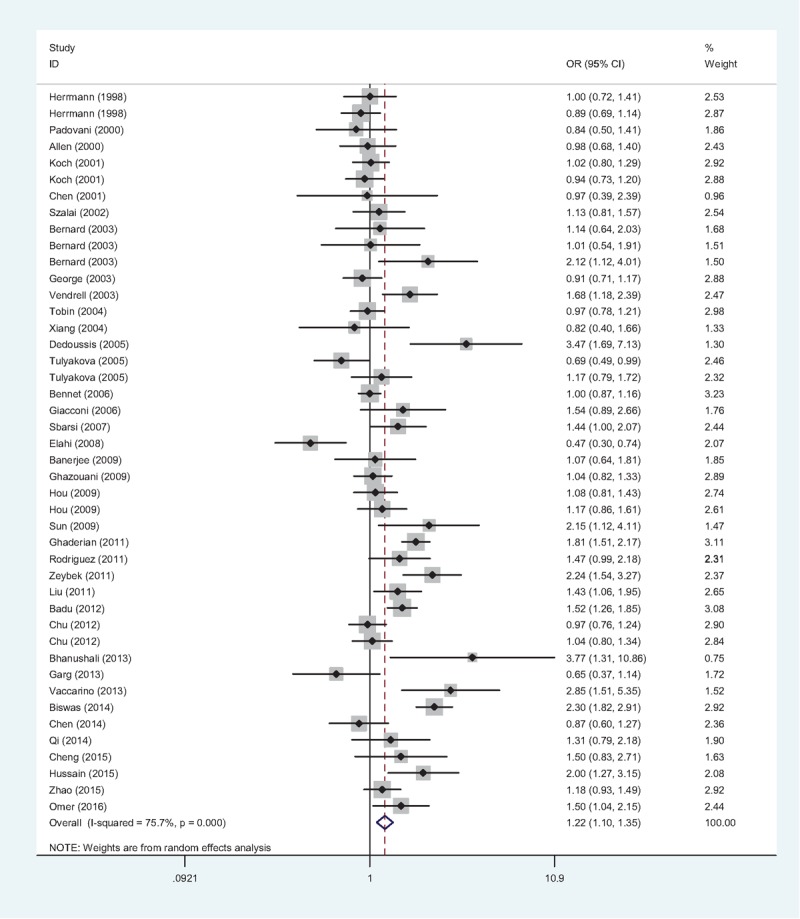
Forest plot of association between TNF-α −308G/A gene polymorphism (A vs G) and IHD risk. The association was indicated as OR with the corresponding 95% CI. The analysis was performed using the STATA 12.0. CI = confidence interval, IHD = ischemic heart disease, OR = odds ratio, TNF-α = tumor necrosis factor-alpha.

Obvious heterogeneity between studies were observed (A vs G: *P* < .001, *I*^2^ = 75.7%; (AA + GA) vs. GG: *P* < .001, *I*^2^ = 81.6%; AA vs (GA + GG): *P* < .001, *I*^2^ = 50.1%), thus the random-effects model were chosen in both 3 models. The pooled ORs revealed a significant association between TNF-α −308G/A gene polymorphism and IHD risk in allele model (A vs G: OR = 1.22, 95% CI = 1.10–1.35, Fig. 3), dominant model ((AA + GA) vs GG: OR = 1.18, 95% CI = 1.03–1.36), and recessive model (AA vs (GA + GG): OR = 1.37, 95% CI = 1.08–1.75), indicating that the TNF-α −308A allele might be an important risk factor for IHD.

As presented in Table 2, in the subgroup analysis by ethnicity, the association between TNF-α −308G/A gene polymorphism and IHD risk was confirmed in both Caucasians (A vs G: OR = 1.23, 95% CI = 1.07–1.43) and Asians (A vs G: OR = 1.20, 95% CI = 1.06–1.35), but not in Indians (A vs G: OR = 1.48, 95% CI = 0.98–2.24). When analyzing the included studies according to the sample size, the group with the sample size < 600 (A vs G: OR = 1.29, 95% CI = 1.08–1.54) showed a more significant association than the group with the sample size ≥600 (A vs G: OR = 1.16, 95% CI = 1.02–1.32). Further subgroup analysis by genotyping method and control source showed that, the association were only confirmed in the studies using PCR-RFLP (A vs G: OR = 1.27, 95% CI = 1.09–1.48) and hospital-based studies (A vs G: OR = 1.29, 95% CI = 1.07–1.56). Furthermore, in the subgroup analyses by matching method (A vs G: OR = 1.32, 95% CI = 1.13–1.54), quality (A vs G: OR = 1.23, 95% CI = 1.11–1.37), and HWE (A vs G: OR = 1.14, 95% CI = 1.04–1.25), the results demonstrated that the TNF-α −308A allele was likely to be related to an increasing risk for IHD in the well-designed studies.

**Table 2:**
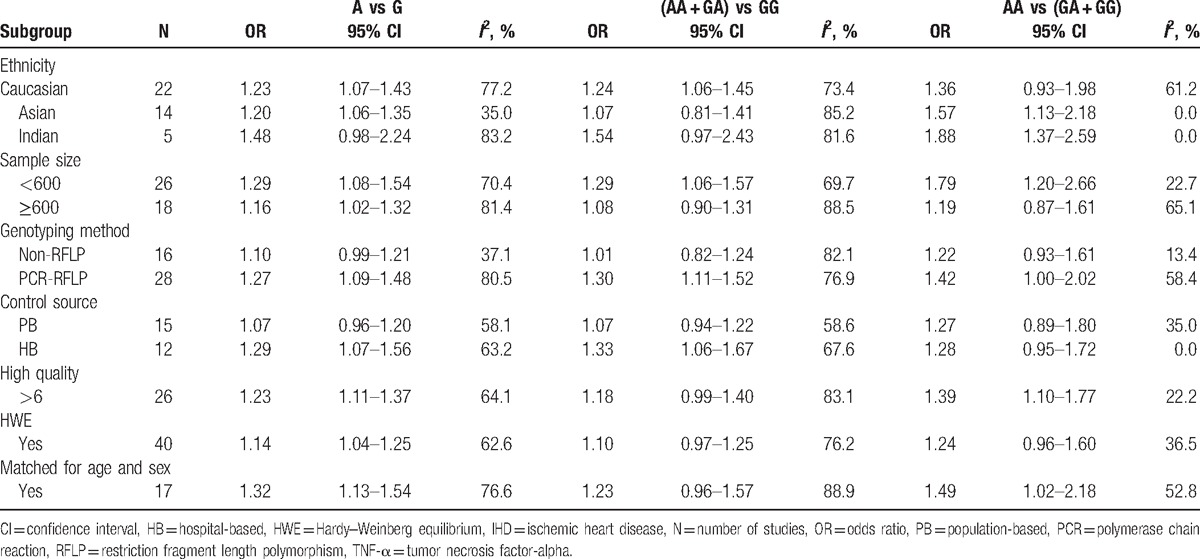
Results of subgroup analysis of association between TNF-ɑ −308G/A gene polymorphism and IHD risk.

#### 3.3.3 TNF-α −857C/T, −863C/A, and −1031T/C

**Figure 4:**
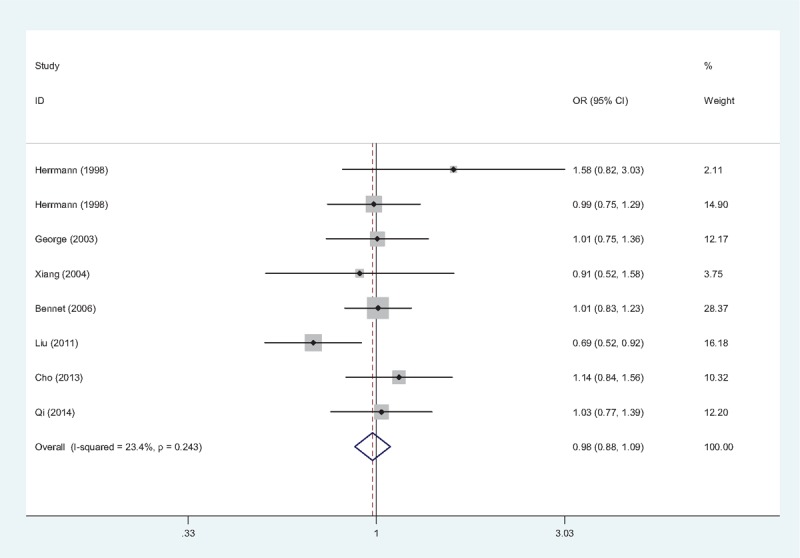
Forest plot of association between TNF-α −857C/T gene polymorphism (T vs C) and IHD risk. The association was indicated as OR with the corresponding 95% CI. The analysis was performed using the STATA 12.0. CI = confidence interval, IHD = ischemic heart disease, OR = odds ratio, TNF-α = tumor necrosis factor-alpha.

**Figure 5:**
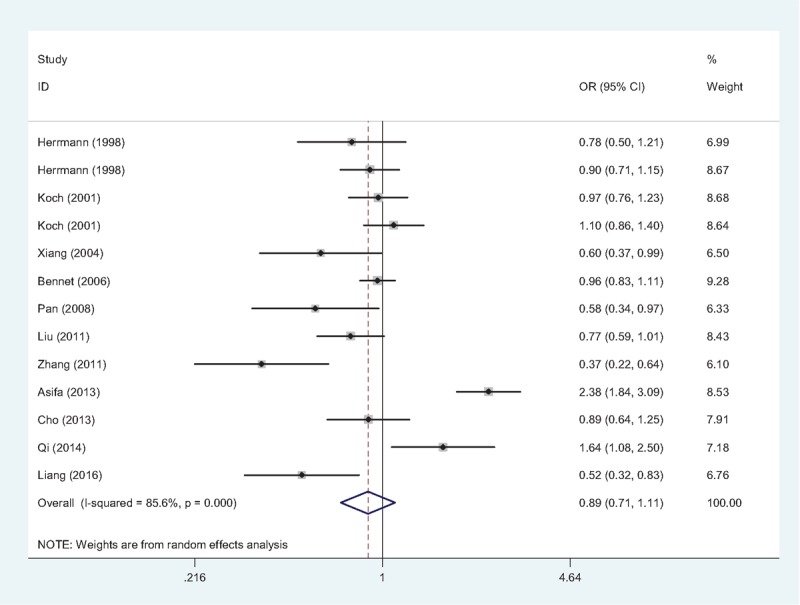
Forest plot of association between TNF-α −863C/A gene polymorphism (A vs C) and IHD risk. The association was indicated as OR with the corresponding 95% CI. The analysis was performed using the STATA 12.0. CI = confidence interval, IHD = ischemic heart disease, OR = odds ratio, TNF-α = tumor necrosis factor-alpha.

**Figure 6:**
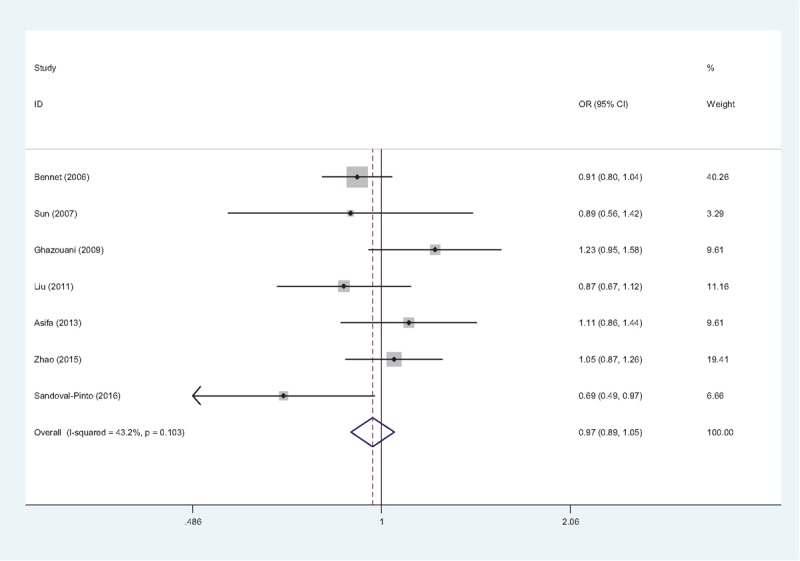
Forest plot of association between TNF-α −1031T/C gene polymorphism (C vs T) and IHD risk. The association was indicated as OR with the corresponding 95% CI. The analysis was performed using the STATA 12.0. CI = confidence interval, IHD = ischemic heart disease, OR = odds ratio, TNF-α = tumor necrosis factor-alpha.

Based on the results of heterogeneity test, different effect models were chosen in the meta-analyses of the 3 polymorphisms. The pooled ORs demonstrated a lack of association between TNF-α −857C/T (T vs C: OR = 0.98, 95% CI = 0.88–1.09, Fig. 4; (TT + CT) vs. CC: OR = 0.95, 95% CI = 0.84–1.07; TT vs (CT + CC): OR = 1.21, 95% CI = 0.88–1.66), −863C/A (A vs C: OR = 0.89, 95% CI = 0.71–1.11, Fig. 5; (AA + CA) vs. CC: OR = 0.89, 95% CI = 0.66–1.20; AA vs (CA + CC): OR = 0.83, 95% CI = 0.63–1.08), −1031T/C (C vs T: OR = 0.97, 95% CI = 0.89–1.05, Fig. 6; (CC + CT) vs. TT: OR = 0.95, 95% CI = 0.86–1.05; CC vs (CT + TT): OR = 0.95, 95% CI = 0.62–1.45) gene polymorphisms and IHD risk. Subgroup analyses were conducted, while no association was observed in any subgroup of these 3 polymorphisms (data not shown).

### 3.4 Publication bias and sensitivity analysis

The potential publication bias of included studies were assessed using the Begg rank correlation test and Egger linear regression test. No significant publication bias were found in the Begg test and Egger test. Furthermore, the funnel plots did not show asymmetrically, which indicating absence of publication bias. Sensitivity analysis was conducted by removing one study each time to observe the influence of each included study on the overall pooled OR. No single study was found to significantly influence the overall pooled OR, which indicated our results were stable.

## 4 Discussion

The association between the TNF-α gene polymorphisms and IHD risk had been highly controversial during the past decades. A previous meta-analysis carried out by Pereira et al^[57]^ in 2007 failed to confirm the association between the TNF-α −308G/A gene polymorphism and IHD risk, while the results of the meta-analysis performed by Wang et al^[2]^ in 2015 were not consistent with Zhang, indicating that the variant allele −308A was positively related to an increasing risk of IHD in total population. Furthermore, there was no other meta-analysis on the relationship between the other TNF-α gene polymorphisms and IHD risk. Therefore, a systematic review and meta-analysis aiming to assess the role of TNF-α gene polymorphisms in the risk of IHD on all current published studies was conducted in the present study.

Pooled on all the available evidence up to date, our meta-analysis, on the basis of 94 independent studies with 17,375 cases and 15,375 controls involved, suggested that the TNF-α −308G/A gene polymorphism was significantly associated with IHD risk. According to the allele model, the variant allele −308A presented a 1.22-fold higher risk of developing IHD compared to the wild allele −308G. Besides, no statistical relationship was found for other TNF-α gene polymorphisms summarized in this systematic review and meta-analysis. No significant publication bias were found. Sensitivity analyses indicated that our results were stable.

Subgroup analysis according to ethnicity showed that, the TNF-α −308G/A gene polymorphism appeared to be associated with IHD risk in both Caucasians and Asians, but not in Indians. The most likely reasons lie in the population stratification within involved studies, especially when both allelic frequencies and incidence of disease vary across ethnic groups.^[58]^ In the present study, the A allele frequency of the TNF-α −308G/A among Caucasians, Asians, and Indians were not the same (14.63% in Caucasians, 8.24% in Asians, and 13.88% in Indians). Additionally, insufficient statistical power resulted from the much smaller sample size (only 5 studies with 1598 cases and 1499 controls were in Indians) could also help to interpret the distinct results from populations with different genetic backgrounds.^[58,59]^

We conducted subgroup analysis by genotyping method because of the increasing demand for precise diagnoses with advanced genotyping method.^[60]^ There were 28 out of 44 studies using PCR-RFLP and 16 studies using other methods such as PCR-SSCP, MS, and TaqMan. Similarly to the total population, significant association between TNF-α −308G/A gene polymorphism and IHD risk was observed in the PCR-RFLP studies rather than the non-RFLP studies, suggesting that PCR-RFLP might be a good choice for genotyping method in DNA polymorphism analysis. Hence, when analyzing DNA polymorphism, we suggested using PCR-RFLP as a good choice of genotyping method according to the DNA sample size and the number of SNPs.^[60]^

In the subgroup analysis by control source, a significant association was observed among hospital-based studies only but not among population-based studies. This finding might due to the selection bias of the hospital-based case–control studies, because such controls might fail to represent the general population absolutely.^[61]^ Therefore, the selection bias should be avoided in the case–control studies as far as possible. In addition, in a further subgroup analysis by sample size, a more significant association between TNF-α −308G/A gene polymorphism and IHD risk was observed in the group with the sample size <600 than in the group with the sample size ≥600. As described in other studies, small sample size studies were more likely to overestimate the effect of the genetic factors.^[62]^ Thus, future well-designed studies with larger samples are needed.

Compared with the meta-analyses conducted by Pereira et al^[57]^ (including 17 studies in 15 articles) and Wang et al^[2]^ (including 36 studies in 28 articles) only analyzing the association between TNF-α −308G/A gene polymorphism and IHD risk, our study had a more sufficient power to investigate the role of TNF-α gene polymorphisms in the risk of IHD, for 45 articles with 32,750 participants were involved. Except for the TNF-α −308G/A gene polymorphism, we also investigated the polymorphisms on the other position of TNF-α gene published up to date. What's more, we performed subgroup analysis by ethnicity, quality, HWE, control source, matching method, sample size, and genotyping method in the present study, which could help to identify the potential sources of heterogeneity.

Finding a certain link between inflammatory genetic markers and IHD requires a number of well-designed studies of phenotypically homogeneous subjects as well as multiple analyses of gene–gene and gene–environment interactions.^[57]^ According to our study, it is believable that TNF-α −308G/A gene polymorphism might be a suitable genetic marker for IHD risk. However, its presence under special environment circumstances (such as psychological stress) might be a key risk factor for IHD susceptibility.^[63]^ As described, the TNF-α genes were located within the region of highly polymorphic variation MHC and they were in linkage disequilibrium with each other and other genes, making the genetic influences caused by genes in linkage disequilibrium could not be avoided.^[59]^ Hence, additional method such as haplotypic analysis with other genetic markers may provide more useful data for the study of genetic etiology of IHD than current available single genotype-based data.

Although some credible findings had been achieved, some limitations of our meta-analysis should be noted here. Firstly, the number of some included studies and the total sample size of some polymorphisms like −1031T/C polymorphism were relatively small, which restricted the statistical power for calculating a more accurate estimate about the association between TNF-α gene polymorphisms and IHD risk. Secondly, the ORs of all included studies were on the basis of unadjusted estimate, as not each included study reported an adjusted OR. Lastly, there was a lack of relevant studies about the association in Africans, Indians, and other ethnic groups, which restricted the power for finding the potential differences in the different ethnicities.

In conclusion, despite the limitations, the present systematic review and meta-analysis indicated a possible association between the TNF-α −308G/A gene polymorphism and IHD risk, demonstrating that the −308A allele might be a risk factor for IHD. However, evidence was limited to confirm the role of TNF-α −238G/A, −857C/T, −863C/A, −1031T/C and other TNF-α gene polymorphisms in the risk of IHD. In order to confirm the current conclusion, future well-designed studies with larger samples are needed.

## Acknowledgment

We highly appreciated the support from all the participants.
